# Longitudinal decline in structural networks predicts dementia in cerebral small vessel disease

**DOI:** 10.1212/WNL.0000000000005551

**Published:** 2018-05-22

**Authors:** Andrew J. Lawrence, Eva A. Zeestraten, Philip Benjamin, Christian P. Lambert, Robin G. Morris, Thomas R. Barrick, Hugh S. Markus

**Affiliations:** From the Stroke Research Group (A.J.L., H.S.M.), Clinical Neurosciences, University of Cambridge; Neurosciences Research Centre (E.A.Z., P.B., C.P.L., T.R.B.), Molecular and Clinical Sciences Research Institute (E.A.Z., P.B., C.P.L., T.R.B.), St George's University of London; and Department of Psychology (R.G.M.), King's College Institute of Psychiatry, Psychology and Neuroscience, London, UK.

## Abstract

**Objective:**

To determine whether longitudinal change in white matter structural network integrity predicts dementia and future cognitive decline in cerebral small vessel disease (SVD). To investigate whether network disruption has a causal role in cognitive decline and mediates the association between conventional MRI markers of SVD with both cognitive decline and dementia.

**Methods:**

In the prospective longitudinal SCANS (St George's Cognition and Neuroimaging in Stroke) Study, 97 dementia-free individuals with symptomatic lacunar stroke were followed with annual MRI for 3 years and annual cognitive assessment for 5 years. Conversion to dementia was recorded. Structural networks were constructed from diffusion tractography using a longitudinal registration pipeline, and network global efficiency was calculated. Linear mixed-effects regression was used to assess change over time.

**Results:**

Seventeen individuals (17.5%) converted to dementia, and significant decline in global cognition occurred (*p* = 0.0016). Structural network measures declined over the 3-year MRI follow-up, but the degree of change varied markedly between individuals. The degree of reductions in network global efficiency was associated with conversion to dementia (B = −2.35, odds ratio = 0.095, *p* = 0.00056). Change in network global efficiency mediated much of the association of conventional MRI markers of SVD with cognitive decline and progression to dementia.

**Conclusions:**

Network disruption has a central role in the pathogenesis of cognitive decline and dementia in SVD. It may be a useful disease marker to identify that subgroup of patients with SVD who progress to dementia.

Cerebral small vessel disease (SVD) is the most common pathology underlying vascular dementia. Characteristic MRI appearances include white matter hyperintensities (WMH), lacunar infarcts, cerebral microbleeds (CMBs), and diffuse white matter damage on diffusion tensor imaging (DTI). Each is individually associated with cognition, but how these associations result in dementia is incompletely understood.^1,2^ A popular hypothesis is that cognitive impairment results from disconnection of cortical-subcortical and cortical-cortical connections,^3–5^ leading to disruption of large-scale brain networks underlying cognitive domains affected by SVD, such as executive function and processing speed.^3^ This hypothesis implies that network connectivity will mediate associations between conventional SVD brain pathologies and cognitive impairment.

Structural brain networks can be measured in humans using magnetic resonance (MR) tractography, derived from DTI data.^6^ Cross-sectional studies suggest network disruption has an important role in cognitive impairment in SVD,^5,7,8^ and have shown reduced network integrity at baseline predicts future dementia. However, such association data cannot prove causality, and there are no longitudinal data showing whether change in MR network parameters over time predicts change in cognition and future dementia risk.

In the St George's Cognition and Neuroimaging in Stroke (SCANS) Study, patients with symptomatic SVD were followed up with annual MRI for 3 years and cognitive testing for 5 years. We determined whether longitudinal change in network measures predicted future dementia and cognitive decline. We also determined whether change in network measures mediated the association between conventional MRI markers of SVD and cognitive decline, to investigate whether network disruption might have a causal role in cognitive decline.

## Methods

### Standard protocol approvals, registrations, and patient consents

The study was registered (ukctg.nihr.ac.uk; study ID: 4577) and approved by a local research ethics committee (London–Wandsworth). Participants provided written informed consent.

### Study participants

One hundred twenty-one patients were recruited between 2007 and 2010 from stroke services at 3 hospitals covering a geographically contiguous region of South London.

Inclusion criteria were a clinical lacunar stroke syndrome^9^ with MRI evidence of an anatomically appropriate lacunar infarct (defined as a high-signal lacunar infarct on diffusion-weighted imaging or a cavitated lacune on T1-weighted [T1w] imaging of maximum diameter ≥1.5 cm), in addition to confluent WMH of Fazekas grade 2 (early confluent) or higher.^10^ The following exclusion criteria were applied: (1) any other stroke mechanism including intra/extracranial large artery stenosis >50%, cardioembolic source, subcortical infarcts >1.5 cm in diameter as these are often embolic, or any cortical infarcts; (2) history of major neurologic or psychiatric condition excepting depression; (3) nonfluent in English; (4) not suitable for MRI; and (5) unable to give informed consent.

### Study design

In this prospective cohort study, participants completed 1 baseline and 3 annual follow-up assessments comprising clinical assessment, MRI, and cognitive assessment. Subsequently, 2 further annual cognitive assessments were conducted.

Baseline assessments were conducted a minimum of 3 months after the most recent stroke to reduce the influence of acute ischemia on MRI and cognition measures. Those who experienced a subsequent clinical stroke could remain in the study provided the new stroke was lacunar.

We report results from participants with MRI follow-up data. Of 121 patients recruited, 103 attended more than one assessment. Eighteen completed only one assessment because of death (n = 7), study withdrawal (n = 6), relocation (n = 1), lost to follow-up (n = 2), or withdrawal from full neuropsychological testing (n = 2). Of the 103 participants with follow-up, MRI follow-up was available for 99. Two further participants had technically inadequate diffusion MRIs, leaving 97 included in this analysis.

As previously described,^11,12^ not all of these participants completed all annual assessments: 20/97 had 12-month MRI follow-up and 11/97 had 24-month MRI follow-up. A total of 249.4 person-years were observed for MRI and 376.1 person-years for cognitive assessments. The mean ± SD duration of MRI follow-up was 2.57 ± 0.84 years, and 5th and 95th percentiles were 1.01 and 3.38 years; for cognitive follow-up, these were 3.88 ± 1.55 years, and 5th and 95th percentiles were 1.00 and 5.23 years. Linear mixed models were used to account for variability in time of assessment and missing data.

### Cognitive assessment

A battery of well-established, standardized tasks sensitive to the cognitive impairments seen in SVD was administered. Full details have been published previously.^11,13^ Tasks are described in table e-1 (links.lww.com/WNL/A463). Task performance was converted to an age-scaled *z* score using the participant's baseline age and published normative data. We analyzed the mean average of these scores as an index of global cognitive function.

### Conversion to dementia

Dementia was diagnosed using the DSM-V^14^ definition of *major neurocognitive disorder* and was present if individuals met at least one of the following criteria:

1. A diagnosis of dementia made in a memory clinic or equivalent clinical service.
2. After review of medical records and cognitive assessments by a neurologist and clinical neuropsychologist who were both blind to all MRI and risk factor information and who both agreed that the clinical picture met DSM-V criteria for dementia.
3. A Mini-Mental State Examination score consistently <24, indicative of cognitive impairment^15^ and reduced capabilities in daily living as measured by a score ≤7 on the Instrumental Activities of Daily Living.^16^

In all cases, the presence or absence of dementia was determined by consensus between a neurologist (H.S.M.) and clinical neuropsychologist (R.G.M.) blinded to participant identity and to the MRI results.

### MRI acquisition

A 45-minute multimodal, whole-brain MRI protocol was acquired using a 1.5T Signa HDxt MRI system (General Electric, Milwaukee, WI) with maximum gradient amplitude of 33 mT/m and a proprietary head coil. The following whole-brain sequences were obtained: axial fluid-attenuated inversion recovery (FLAIR), coronal spoiled gradient recalled echo 3-dimensional T1w, and gradient recalled echo T2*-weighted sequence. Full acquisition details have been previously published.^5,13^ There were no upgrades to the scanner or software over the course of the study.

### Diffusion acquisition and preprocessing

Diffusion data comprised axial single-shot diffusion-weighted spin-echo planar imaging with isotropic resolution (2.5 mm^3^) and 25 diffusion gradient directions at b = 1,000 s/mm^2^ in positive and negative gradient directions. Eight echo planar images (EPIs) were acquired without a diffusion gradient (b = 0 s/mm^2^). These images were coregistered and the average was taken to give a T2-weighted EPI, which we term the “b0” image.

Diffusion preprocessing has been described in full previously.^5^ In brief, diffusion-weighted images were realigned to remove eddy current distortions, and slices with signal loss caused by motion were identified and excluded from further analysis. Diffusion-weighted volumes with opposite gradients were geometrically averaged to eliminate gradient cross-terms. Diffusion tensors were then fitted using the least-squares method.

### Longitudinal MRI processing

We applied an image registration pipeline, optimized for longitudinal research, which reduces bias induced by independently processing images at each time point.^17^ For each participant, longitudinal template images were independently created for 2 MR modalities: T1w anatomical images, and b0 EPI from the diffusion sequence. Templates were constructed using the buildtemplateparallel.sh tool (v0.0.14),^18^ part of the Advanced Normalization Tools software package (stnava.github.io/ANTs/).^19^ Two participants were excluded from analysis because of a failure of this processing pipeline to accommodate large magnetic susceptibility-related distortions in the diffusion acquisitions.

### Tissue segmentation and WMH measurement

Coregistered T1w and FLAIR images were segmented into gray matter, normal-appearing white matter, CSF, and WMH tissue components using a 2-step technique adapted and optimized to our population and previously described.^20,21^ In brief, T1w and FLAIR images were coregistered and an initial joint segmentation was conducted, followed by warping to a group template. Tissue probability maps in this group space were then calculated and used to refine segmentations in native space. Finally, an extraventricular CSF tissue class was manually defined (including cavitated lacunes and enlarged Virchow-Robin spaces) and used to generate repaired tissue maps for gray matter, normal-appearing white matter, CSF, and WMH.

### Conventional MRI markers of SVD

#### Brain volume

Tissue segment volumes were calculated from the tissue segmentation maps calculated above. In this study, we analyzed total parenchymal brain volume.

#### Hippocampus volume

Jacobian determinant images were calculated from the transformations to the standard space template^21^ described above. Hippocampal regions of interest (ROIs) from the automated anatomical labeling (AAL) atlas^22^ were used to mask the jacobian images and the jacobian determinants summed to give the total degree of expansion or contraction in the hippocampus for each participant. Multiplying this quantity by the volume of the ROI produces the estimated hippocampal volume.

#### White matter hyperintensities

WMH were assessed by the total volume of the WMH tissue class. To adjust for the effects of head size and brain atrophy on lesion volume, we analyzed WMH load by expressing WMH volume as a fraction of the volume of total white matter.

#### Lacunes of presumed vascular origin

A consultant neuroradiologist evaluated T1w and FLAIR images for cavitated lacunes of presumed vascular origin.^2^ Lacunes were defined as CSF-filled cavities 3 to 15 mm in diameter, with a surrounding rim of FLAIR hyperintensity.^2^ Based on the results of previous studies,^13,23^ we analyzed the log_10_-transformed total lacune count.

#### Cerebral microbleeds

CMBs were identified on gradient echo images using the Brain Observer MicroBleed Rating Scale as previously described.^13^ All baseline CMBs were identified by a single consultant neuroradiologist. Presence and number of new CMBs in follow-up images were identified by a single trained rater. Based on the results of previous studies,^13,23^ we analyzed the log_10_-transformed total CMB count.

#### DTI measures

The diffusion tensor was decomposed into maps of directional invariants fractional anisotropy (FA) and mean diffusivity (MD). Histogram analysis was then applied (as previously described^12^) to provide whole-brain measures of FA and MD in white matter. In previous work, we found normalized peak height (NPH) of the MD histogram to be the most sensitive measure of ultrastructural change in SVD.^12^ Therefore, we restricted our analysis of DTI data to this measure. The NPH of the MD histogram represents the fraction of the white matter that falls into the peak bin of the histogram. Larger values of MD-NPH indicate that a greater proportion of white matter holds healthy MD values.

### Network construction

Brain network construction requires the definition of nodes (brain regions) and edges (connections).

#### Network nodes

Network nodes were defined using the AAL atlas^22^ of 90 regions (45 bilateral regions, comprising 40 cortical and 5 subcortical), labeled on the Colin27 T1w image. To minimize registration error to this atlas, we constructed an intermediate study-specific T1w template, using subject-template T1w images, as follows. First, signal intensity in regions of white matter lesion in the T1w images were repaired using the SLF toolbox (atc.udg.edu/nic/slfToolbox).^24^ Second, repaired images were iteratively registered to create an initial study-specific template.^18^ The resulting group template image was then registered to the Colin27 T1w image.

Finally, node ROIs were defined by applying a single composite transform bringing the AAL labels through the following transformation spaces: Colin27 → Group Template (T1w) → Subject Template (T1w) → Subject Template (b0-epi) → native time point (b0-epi). Labels were transformed using multilabel gaussian interpolation.

#### Network edges

Whole-brain deterministic tractography was applied to the diffusion tensor using the method of Basser as previously described.^13^ In brief, streamlines were seeded on a super-resolution 0.5-mm^3^ grid where FA ≥0.2. Step size was 0.5 mm and termination criteria were FA <0.2 and angle >45.0°. The streamlines were length thresholded between 20 and 250 mm.

Network edges were defined for each pair of atlas regions (A, B) where one or more streamlines terminated with one end in A and another in B. Weights were calculated for each edge from the number of streamlines,^5^ modified from Hagmann et al.,^6^ with adjustments to correct for distance traveled and the seeding scheme as follows: for each streamline connecting 2 ROI pairs, the inverse length (in millimeters) was calculated and summed. The sum of such inverse lengths was divided by 2 to correct for the number of seeds per millimeter of streamline length. To reduce the effects of low-weight, false-positive connections, a threshold (weight ≥1) was adopted. We omit adjustment of each edge weight for the average volume of ROIs as, for a given tract size, this would over-weight connections between smaller unimodal regions and correspondingly under-weight the connections of network hubs, which connect to multiple areas.

### Network analysis

For each brain network, network efficiency analysis was applied along with calculation of network properties including number of edges, average edge weight, and total network strength (sum of edge weights) using the brain connectivity toolbox (brain-connectivity-toolbox.net).^25^ Our analyses focus primarily on network global efficiency, because this captures topological network information and has shown promise in previous investigations of SVD.^5,7^

### Statistical analysis

Data processing and analysis was performed in the R language and environment for statistical computing (v3.2.3; r-project.org/). For the analyses described below, all bivariate relationships, and model residuals, were visually inspected and assumptions tested. Variance inflation factors were within commonly accepted limits (<4).

#### Longitudinal change

To study change over time, we used linear mixed-effects regression (LMER) with random effects of intercept and linear slope (with respect to time), with the lme4 package (v1.1-11).^26^ LMER accounts for the hierarchical nature of the data, allowing imbalance, data missing at random, and variability in the timing of assessments.^27^ The fixed effect of time represents the group average annualized change of the variable, which we tested for statistical significance using Satterthwaite approximation to the degrees of freedom. For subsequent analyses, random effects of intercept and slope were extracted for each participant.^27^ This separates results into a baseline score and annualized change while retaining the practical benefits of LMER. This was conducted for all continuously varying variables. For 2 count measures (lacune count, CMB count), we dichotomized change.

#### Correlates of network change

Relationships between the amount of change in the network and conventional MRI variables were explored using multiple linear regression with the network variable as the dependent and MRI variables as predictors. We first explored pairwise (single predictor) relationships and then identified the independent predictors of network change using forward stepwise model selection. Selection steps proceeded on the basis of minimizing model Akaike information criterion.

#### Relationships with outcome

To assess the relationships between network integrity and dementia, binary logistic regression models were fitted with conversion status the dependent variable. For the global cognitive function index, linear regression models were fitted with annualized change the dependent variable. As above, single variable relationships were estimated first, followed by a stepwise model selection to identify the independent contributions in a best fitting model.

For models with dementia as the dependent variable, linear mixed-effects estimates for predictors were separately calculated to exclude MRI data acquired after conversion to dementia (n = 3 participants, n = 3 observations). This ensured that the MRI data used to predict dementia was acquired before diagnosis of dementia.

Finally, we tested the mediation hypothesis, looking at whether changes in the brain network explained relationships between other MRI variables and the outcome variables. Causal mediation models (analogous to the Sobel test) were estimated using the mediation package for R (v4.4.5)^28^ employing a nonparametric bootstrap with 10,000 samples.

### Data availability

The identified summary data used in this analysis will be shared with other researchers on request via the corresponding author.

## Results

Baseline demographics and clinical descriptives are presented in table 1.

**Table 1:**
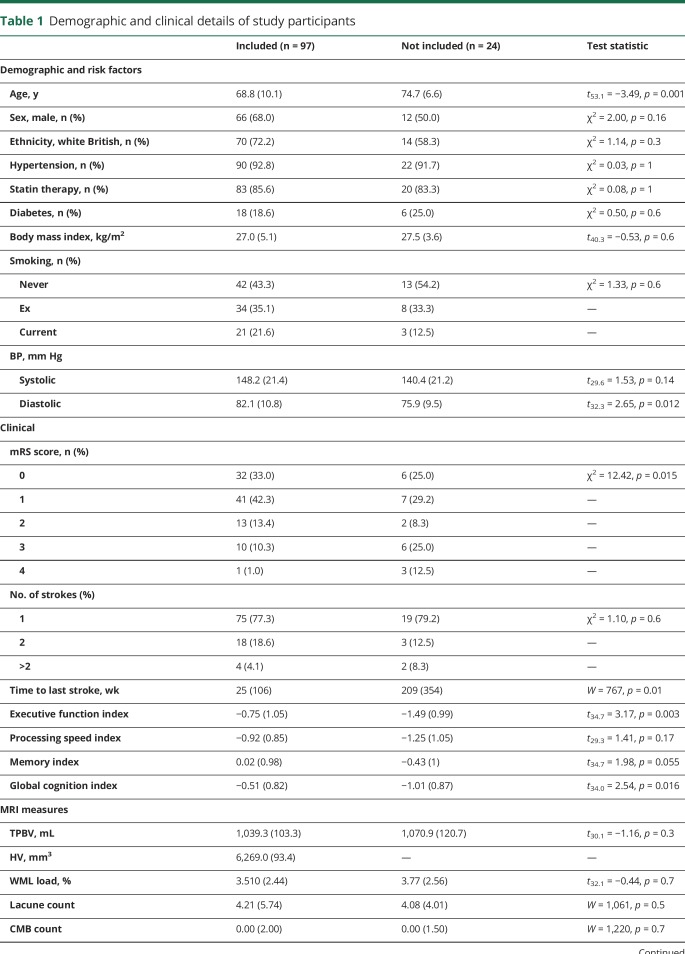

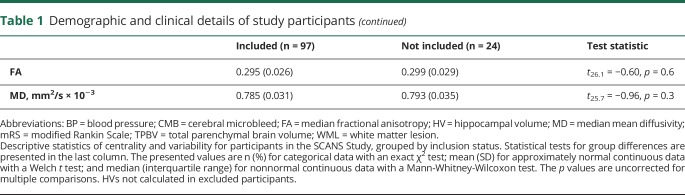
Demographic and clinical details of study participants

### Clinical endpoints during follow-up

During 5-year follow-up, 8 of 97 participants died (8.2%). Causes of death were cancer (n = 1), respiratory (n = 1), intracerebral hemorrhage (n = 2), other health-related cause (n = 2), and not known (n = 1). There were 7 stroke events during follow-up. Four participants experienced new lacunar stroke, of whom 3 withdrew following the stroke because of disability. Three participants experienced intracerebral hemorrhage, of which 2 were fatal as above.

### Outcome measures

There was a significant decline in cognitive test performance over 5 years (table 2). However, cognitive change varied markedly between individual participants, with some showing marked decline and others stable performance.

**Table 2:**
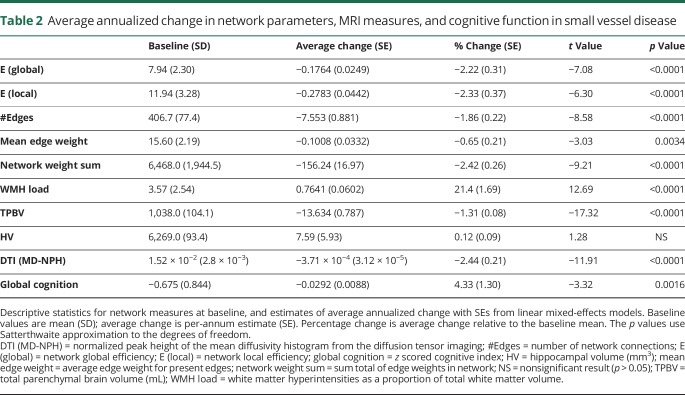
Average annualized change in network parameters, MRI measures, and cognitive function in small vessel disease

Follow-up data on progression to dementia was available for all 97 participants. All participants were dementia-free at baseline. Seventeen (17.5%) of 97 patients converted to dementia during the 5-year follow-up. Dementia diagnosis was based on clinical diagnosis (n = 8), review of medical records (n = 3), and meeting dementia thresholds for Mini-Mental State Examination and Instrumental Activities of Daily Living scores (n = 6). Mean ± SD time to dementia conversion was 3.3 ± 1.4 years.

### Longitudinal change in network measures

**Figure 1:**
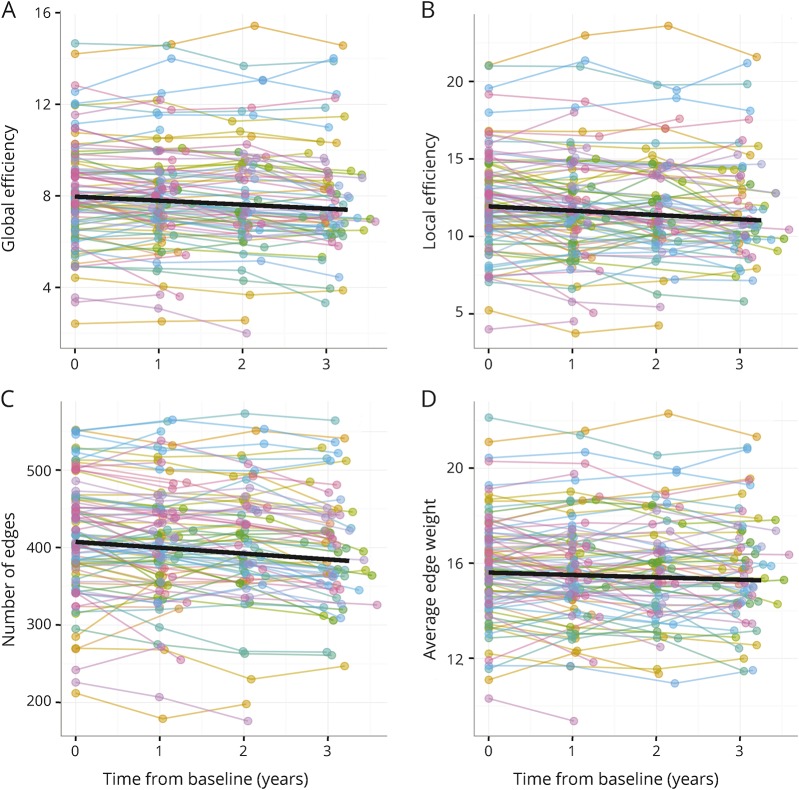
Evolution of network summary measures over time from baseline assessment (A) Network global efficiency, (B) average local network efficiency, (C) number of network edges, and (D) average edge weight (for present edges). Colored circles linked by lines indicate values for individual participants. The black line shows the group average result—the fixed-effect estimates from the linear mixed-effects regression (see table 2).

There was significant decline in all structural network measures during follow-up (table 2). The degree of change in network measures varied markedly between individuals (figure 1). For network global efficiency (baseline mean ± SD = 7.94 ± 2.30), average annual decline was −0.1764, but individual estimates varied between −0.448 and 0.103 consistent with at least some participants having stable network global efficiency.

### Longitudinal change in other MRI markers

Conventional radiologic markers of SVD showed significant change during follow-up (table 2). WMH lesion volume increased, total parenchymal brain volume decreased (indicative of atrophy), and NPH of the MD histogram decreased (indicating a greater proportion of white matter had abnormal diffusivity values). A total of 69 new lacunes were observed on MRI, occurring in 26 of 97 participants. In contrast, only 4 of these were associated with symptomatic lacunar stroke during the study and, of these, one was outside the MRI follow-up period (i.e., between 3 and 5 years). There were 162 new CMBs in 34 of 97 participants. In contrast, no significant changes over time in hippocampal volume were observed; therefore, only baseline values were considered further.

### Prediction of dementia and cognitive decline

A greater decline in network global efficiency over the 3-year MRI follow-up period was significantly associated with both conversion to dementia (B = −2.35, odds ratio [OR] = 0.095, *p* = 0.00056) and with global cognitive decline (β = 0.398, *p* < 0.0001) over the 5-year follow-up period.

Baseline network global efficiency also predicted both dementia and cognitive decline with higher efficiency associated with lower risk of conversion to dementia (B = −1.06, OR = 0.347, *p* = 0.0056) and slower global cognitive decline (β = 0.347, *p* = 0.00076).

Further analysis demonstrated it was the change in network parameters, rather than baseline values, that was driving the associations. The association between baseline score and outcome was no longer significant after controlling for the association with change in network global efficiency (dementia: B = −0.89, OR = 0.41, *p* = 0.08; global cognition: β = 0.16, *p* = 0.17). In contrast, significant associations remained with change in network global efficiency after controlling for baseline network global efficiency (dementia: B = −2.28, OR = 0.10, *p* = 0.0012; global cognition: β = 0.31, *p* = 0.004).

**Figure 2:**
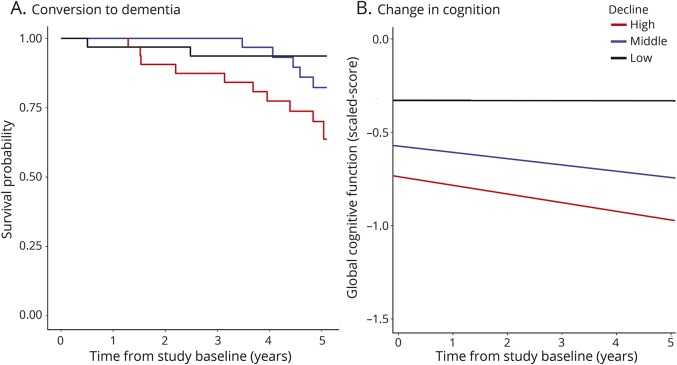
Effects of different amounts of annualized change in network global efficiency on the outcome measures Conversion to dementia (A) and change in global cognition (B). Individuals were divided into 3 equal-sized groups on the basis of the annualized change in network global efficiency (cut points: −0.139, −0.193) with the following median ± interquartile range annualized changes: low decline group (black lines) −0.10 ± 0.05, middle decline group (blue lines) −0.17 ± 0.01, and high decline group (red lines) −0.23 ± 0.06. Separate survival curves (A) or linear mixed model group average intercepts and slopes (B) are plotted for each group.

**Figure 3:**
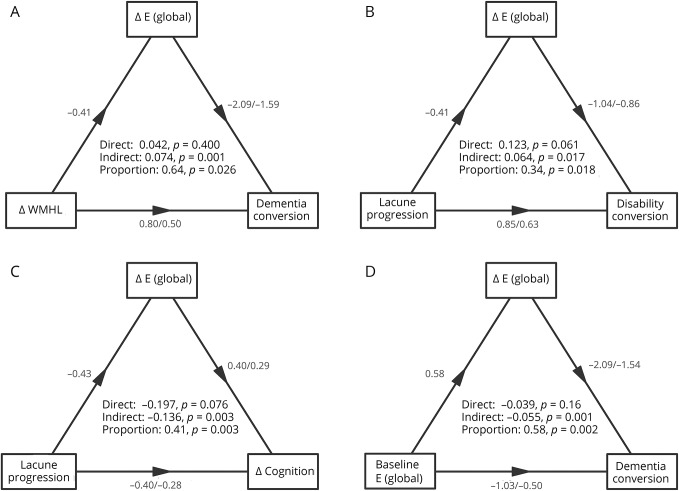
Example path diagrams to illustrate mediation effects In each path diagram, change in network global efficiency (ΔE [global]) is considered as a mediator (top box) of significant relationships. Coefficients associated with the paths (arrows) represent the standardized regression coefficients in the form simple/joint, where “simple” is the coefficient value in the absence of the other variable, and “joint” is the value in the model that includes both predictor and mediator. The results from the statistical mediation analysis (table 4) are included in the center of the figure. The indirect path indicates the significance of the mediation effect on the predictor, the middle value for the indirect path indicates effect of the predictor independent of mediation, and the lower value for the proportion reflects mediation as a fraction of the sum of direct and indirect effects. WMHL = white matter hyperintensity load.

Given this pattern of results, we subjected this relationship to mediation analyses and found a statistically significant proportion of the association between baseline network global efficiency and outcome was mediated by subsequent changes in network global efficiency (dementia: 0.50 [0.22, 1.04], *p* < 0.0001 (figure 2); cognitive function: 0.48 [0.10, 0.99], *p* = 0.012). Figure 3 illustrates these effects by dividing participants into 3 groups according to the amount of annualized change in network global efficiency (low, middle, and high change). The proportion of the sample converting to dementia was related to the degree of network decline: low change (n = 1/32, 3%), middle change (n = 4/32, 12.5%), high change (n = 11/33, 34%). For global cognition, the average fitted slopes for middle network decliners (−0.034) were closer to the high network decliners (−0.046) than the low network decliners (−0.0002).

### Comparing network global efficiency with other MRI measures as predictors of dementia and cognitive decline

In table 3, we display associations with outcome for network global efficiency and for conventional MRI measures. On univariate analysis, multiple MRI markers were associated with progression to dementia and cognitive decline (table 3). However, on multivariable analysis, the only independent associations were change in network global efficiency for progression to dementia, and change in network global efficiency and both baseline lacune count and lacune progression for cognitive decline.

**Table 3:**
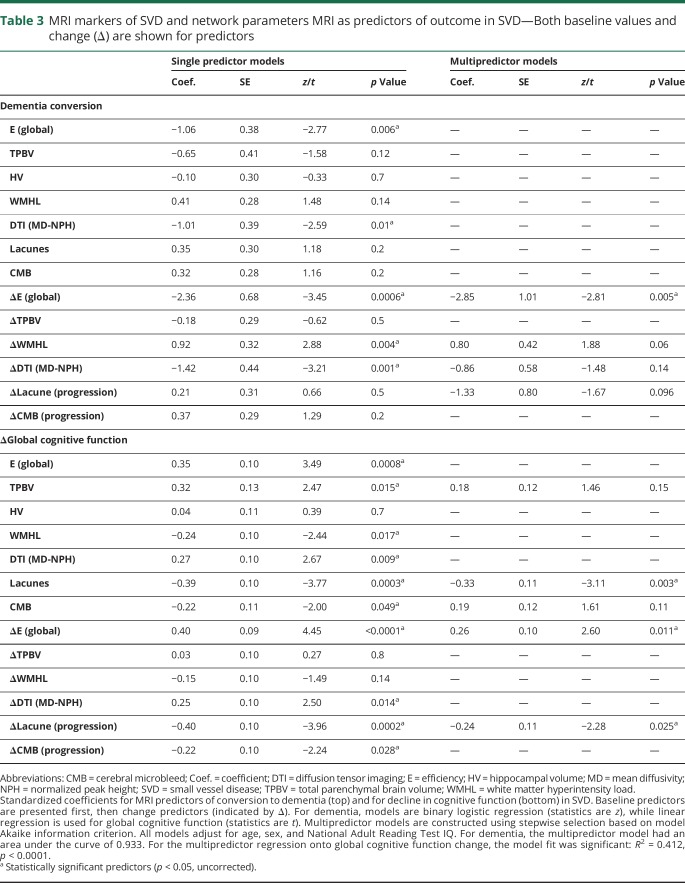
MRI markers of SVD and network parameters MRI as predictors of outcome in SVD—Both baseline values and change (Δ) are shown for predictors

### Investigating mediation via changes in network global efficiency

We assessed whether change in network global efficiency mediated the relationship between change in conventional MRI features of SVD and outcome. Table 4 shows estimated parameters from mediation analyses, with example path diagrams presented in figure 3. Mediation analysis was performed for all MRI variables significantly associated with outcomes in the single predictor models (table 3).

**Table 4:**
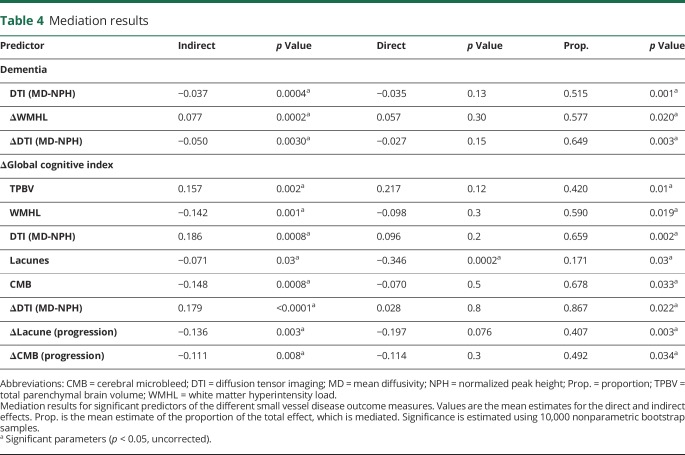
Mediation results

For all MRI predictors of conversion to dementia and change in global cognitive function, a significant proportion of the total effect was mediated by changes in network global efficiency. Only one direct effect, indicating the effect of the predictor after controlling for changes in network global efficiency, was significant—that of the baseline number of lacunes for changes in global cognitive function (table 4).

## Discussion

In this prospective cohort of patients with symptomatic SVD, we were able to detect change in structural networks, with a significant decline in all network parameters, over a 3-year follow-up period during which annual MRI was performed. The rate of decline in network global efficiency independently predicted conversion to dementia and cognitive decline over the 5-year follow-up period. Associations observed for other MRI markers, particularly WMH volume and lacunes, were mediated by their relationship with network changes. Our data are consistent with network disruption having a central role in the mechanism of cognitive decline in vascular cognitive impairment due to SVD. Furthermore, it suggests the rate of change in network global efficiency may be a useful marker to predict risk of progression to dementia.

We observed a wide range of trajectories of cognitive decline. Some participants experienced no or minimal cognitive decline, while others had a rapid decline with progression to dementia; the overall dementia rate was approximately 20% over the 5-year follow-up period. This wide range of cognitive outcomes emphasizes the need for techniques to identify which patients are likely to progress to dementia, both to provide risk prediction information and to identify high-risk individuals for therapeutic interventions. Our results demonstrate that decline in network global efficiency predicts progression to dementia, independently of common MRI markers, and thus may allow identification of that subset of individuals who are at risk of dementia. These findings suggest that the structural network acts as an intermediate (a mediator) between multiple MRI correlates of SVD and clinical outcome. Network measures may be a useful way of integrating information from multiple MRI parameters of SVD into a single predictive score.

Our work adds to the literature by showing that the rate of change in network global efficiency is the primary driver of network decline. Although associations were also found with baseline network global efficiency, these disappeared once the changes were controlled for. This suggests that the association between outcome and baseline network global efficiency is likely attributable to more severe disruption at baseline being a marker of an increased risk of more rapid progression. Of note, in this study in those who progressed to dementia while MRI follow-up was continuing, we included only network measures prior to the diagnosis of dementia in our predictive models, and therefore showed that change in network global efficiency before onset of dementia predicted dementia risk.

In previous cross-sectional investigations of SVD, measures of network global efficiency have been shown to mediate the relationship between MRI markers of SVD and the severity of cognitive impairment.^5,7^ However, differentiating causality from association is impossible in cross-sectional studies. Prospective longitudinal studies in which one can determine whether change in one parameter predicts change in another provide stronger support for a causal relationship. Our longitudinal findings support the hypothesis that conventional MRI markers of SVD (such as white matter lesions and lacunar infracts) cause cognitive decline via disruption of complex brain networks.

Future research could be improved by using MRI with higher field strength and spatial resolution with isotropic voxel dimensions for all sequences.^29^ However, a major strength of the study was highly consistent data obtained from the same scanner without upgrade or change over the full data collection period.

We studied a group of individuals who had moderate to severe symptomatic SVD. By our inclusion criteria of taking patients with both definite ischemic (lacunar stroke) and confluent WMH, we aimed to identify patients in whom ischemic processes were likely to be the cause of their SVD and of any cognitive impairment, but our results require replication in participants with less severe SVD. Although we selected a population in whom SVD was likely to be the primary driver of cognitive impairment and dementia, it is well recognized that at post mortem, many such patients have a mixed picture with both vascular and Alzheimer pathology. To assess the potential role of Alzheimer pathology, we measured hippocampal volumes and found this was not a predictor of dementia in our population. This is in contrast to a population with milder SVD, in which baseline hippocampal volumes and network integrity both predicted dementia.^30^ It is possible that the more severe SVD in the SCANS population means this is therefore the predominant pathology causing dementia in this population, while in cases with milder radiologic changes of SVD, coexistent Alzheimer pathology has a relatively more important role.^31^

A weakness of the study was participant withdrawal. The dropout rate was comparable to other longitudinal aging studies^20^; however, patients without complete follow-up were older and more disabled,^32^ which may bias results toward an underestimation of MRI and cognitive progression rates.

We did not study a control group, and therefore cannot be certain that the observed changes are not, to some extent, attributable to the effects of aging. However, the relationship between change in networks and dementia in this sample implies a key role for network disruption in cognitive decline in patients with SVD. Furthermore, the rates of change in the MRI markers we assessed in this study are lower than in a healthy, aging cohort of similar age imaged on the same scanner.^33^

In vivo structural networks derived from tractography have been shown to be reproducible,^34^ but there are limitations.^35^ Spatial resolution limits diffusion tractography to assessment of larger white matter fascicles, and the directionality of connections and their true functional status cannot be inferred from diffusion data. Furthermore, diffusion MRI data are noisy and tractography algorithms display compounded noise the further they travel, meaning connections are systematically more difficult to track (and correspondingly less reproducible) over longer physical distances. Some common anatomical motifs such as crossing/kissing fibers are ambiguous to tractography algorithms, with particular limitations for the deterministic tractography used in this research.^26^ Methodologic improvements to diffusion imaging and tractography algorithms can address these issues to some extent,^36,37^ but many of the improved methods require data from higher-field-strength scanners, or at higher angular resolution or over multiple b-shells, which is not available in this clinical sample. There is the potential to extend our findings by using improved techniques in the future.

This prospective longitudinal study demonstrates that change in network global efficiency is an independent predictor of dementia, confirms the central role of network disruption in the pathogenesis of cognitive decline in SVD, and supports a disconnection hypothesis of dementia in the disease. While network analysis currently requires offline image analysis, MRI to assess network global efficiency may be a useful surrogate marker for future treatment trials in SVD.

## Glossary

AAL: automated anatomical labeling
CMB: cerebral microbleed
DSM-V: *Diagnostic and Statistical Manual of Mental Disorders* (Fifth Edition)
DTI: diffusion tensor imaging
EPI: echo planar image
FA: fractional anisotropy
FLAIR: fluid-attenuated inversion recovery
LMER: linear mixed-effects regression
MD: mean diffusivity
MR: magnetic resonance
NPH: normalized peak height
OR: odds ratio
ROI: region of interest
SCANS: St George's Cognition and Neuroimaging in Stroke
SVD: small vessel disease
T1w: T1-weighted
WMH: white matter hyperintensity
